# Changes in the midgut diverticula epithelial cells of the European cave spider, *Meta menardi*, under controlled winter starvation

**DOI:** 10.1038/s41598-018-31907-3

**Published:** 2018-09-11

**Authors:** Saška Lipovšek, Tone Novak, Franc Janžekovič, Nina Brdelak, Gerd Leitinger

**Affiliations:** 1 Faculty of Medicine, University of Maribor, Taborska ulica 8, 2000 Maribor, Slovenia; 2 Department of Biology, Faculty of Natural Sciences and Mathematics, University of Maribor, Koroška cesta 160, 2000 Maribor, Slovenia; 3 Faculty of Chemistry and Chemical Engineering, Smetanova ulica 17, University of Maribor, 2000 Maribor, Slovenia; 4 Research Unit Electron Microscopic Techniques, Cell Biology, Histology and Embryology, Gottfried Schatz Research Center for Cell Signaling, Metabolism and Aging, Medical University of Graz, Neue Stiftingtalstrasse 6, 8010 Graz, Austria; 5 Faculty of Health Sciences, University of Maribor, Žitna ulica 15, 2000 Maribor, Slovenia

## Abstract

The European cave spider, *Meta menardi*, is among the most common troglophile species inhabiting the cave entrance zone in Europe, where prey is scarce in winter. Spiders feed only if prey is available; otherwise, they are subjected to long-term winter starvation. We carried out a four-month winter starvation of *M. menardi* under controlled conditions to analyze ultrastructural changes in the midgut diverticula epithelial cells at the beginning, in the middle and at the end of the starvation period. We used light microscopy, TEM and quantified reserve lipids and glycogen. The midgut diverticula epithelium consisted of secretory cells, digestive cells and adipocytes. During starvation, gradual vacuolization of some digestive cells, and some necrotic digestive cells and adipocytes appeared. Autophagic structures, autophagosomes, autolysosomes and residual bodies were found in all three cell types. Spherites and the energy-reserve compounds were gradually exploited, until in some spherites only the membrane remained. Comparison between spring, autumn and winter starvation reveals that, during the growth period, *M. menardi* accumulate reserve compounds in spherites and protein granules, and energy-supplying lipids and glycogen, like many epigean, overwintering arthropods. In *M. menardi*, otherwise active all over the year, this is an adaptive response to the potential absence of prey in winter.

## Introduction

The European cave spider, *Meta menardi* (Latreille, 1804) (Araneae, Tetragnathidae) inhabit the twilight zone of most hypogean habitats across Europe. With a relatively large body (length of 10 to 17 mm; males being smaller than females), it appears among the most distinctive animals of the entrance cave sections^1–12^.

According to the classical ecological classification of subterranean animals^13–15^, animals in subterranean habitats are classified into three groups. While trogloxenes are not adapted, and troglobionts are well adapted to the subterranean habitat, troglophiles are intermediate. *Meta menardi* rank among the troglophile species, which either alternate between the epigean and hypogean habitats or live permanently in subterranean habitats. They show some moderate adaptation to the subterranean habitat, such as partly reduced eyes and adaptations to compensate for the lack of visual orientation^10,16,17^, and partly reduced tolerance to temperatures below 0 °C^18,19^. Some among partly adapted species, including *M. menardi*, do not complete their life cycle underground, while others do. Besides, some overwinter in dormancy (own unpublished data), while the others, like *M. menardi*, are active throughout the year. *Meta menardi* lives about two years. The life cycle consists of two ecophases: a hypogean and an epigean ecophase^3,4,7,9,10^. Adults mate in hypogean habitats in spring. In summer, females produce egg-sacs (cocoons). Juveniles hatch in the late autumn or in winter, but stay within the egg-sacs until early spring. Thereafter, the second-instar spiderlings move out of the caves and spread outside by ballooning. They live in epigean habitats until becoming fourth-stage instars, when they return to the hypogean habitat^3,7,9^. Field-collected data showed that *Meta* spiders are preferentially associated with prey-rich areas of caves^9–11^.

For cave spiders prey availability and abiotic features are major determinants of habitat suitability^11^. Specific prey dynamics means only short-term availability of prey for orb-weaving spiders within caves in winter^4^. This is likely the reason that *M. menardi* combine catching flying prey in webs and crawling prey on the cave walls^3,4,9,20–22^.

In spiders, the midgut epithelium consists of four cell types: basal, secretory and digestive cells and guanocytes^23,24^. Basal cells are not differentiated and gradually transform into secretory and digestive cells^23,24^. An abundant rough endoplasmic reticulum, and many electron-dense granules containing digestive enzymes are characteristic of the secretory cells^23,24^ and digestive vacuoles of the digestive cells^23^. Guanocytes are specialized absorptive cells, which metabolize and store nitrogen products like purine, guanine and uric acid^23,24^.

Macroautophagy − referred to as autophagy^25,26^ − is the best studied process. It is an important process in response to starvation^27–29^ and other stress factors, e.g., microsporidian infection of the midgut^30^. In arthropods overwintering in hypogean habitats, autophagy is an important pro-survival process^31,32^. During autophagy, a portion of the cytosol is surrounded by a double-membrane – the phagophore, forming a double-membrane organelle – the autophagosome. When an autophagosome fuses with a lysosome, they form the autolysosome, which is a single-membrane structure, containing electron-dense amorphous material^26^. Thus, the autophagy is a common survival and defensive response in any until recently studied organisms. It is activated by stress factors. However, the autophagy may show a certain variation with respect to sites and abundance in the cell of autophagic structures, which appear during starvation. In the context of our study, both energy and nutrient resources are required in the cell maintenance during long-term starvation and changes in both these resources are of central interest to identify the survival strategy in starving individuals. While either prevalently lipid or prevalently glycogen energy support, as well as graduate spherite exploitation to release nutrients is expected, the specific course of autophagy in these organisms could eventually decover a halfway pattern in adaptation to the subterranean milieu. This could eventually contribute to understanding the evolutionary pathways of spiders to the subterranean habitats−an issue that has been strongly understudied.

In natural habitats in winter, *M. menardi* are active and feed if they catch prey (own, unpublished data). If not, they carry out a kind of natural winter starvation, resembling the programmed starvation in dormant invertebrates in caves (e.g., refs^32–34^). In this respect, *M. menardi* is a model species to study evolutionary steps in the processes of adaptation to life in the subterranean environment. For this reason, we expected *M. menardi* to show an intermediate adaptation with respect to epigean and hypogean spiders. We hypothesized that in starved *M. menardi* the same types of changes appear as in other naturally starved arthropods during overwintering. Hovewer, in different seasons we expected unequal responses to starvation, in contrast to hypothetically uniform response all over the year in well adapted subterranean species. For easier comparison, we use the word overwintering here to mark conditions in starved *M. menardi* in winter. We asked which are characteristic changes in the midgut diverticle epithelial cells, and which energy-supplying compounds *M. menardi* spend during overwintering. Finally, we compared findings in *M. menardi* starved during the growing period with relatively abundant prey^35^, and those starved during the wintertime with much more limited prey availability. This analysis of the whole-year response to starvation in *M. menardi* could eventually decover some specific features in supporting cells with energy and nutrients in the hypothetically stepwise adaptation to subterranen habitats.

## Results

**Figure 1:**
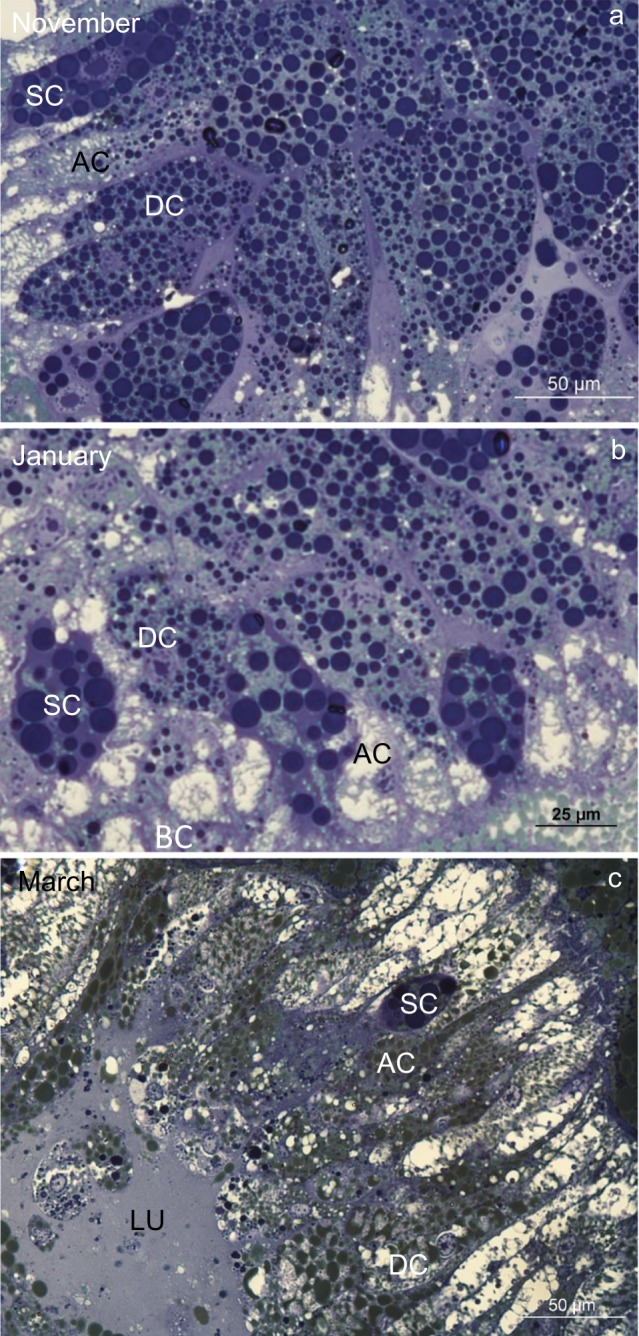
Semithin section of the midgut diverticula of *Meta menardi*. (**a**) The beginning of the experiment (November). (**b**) The middle of the experiment (January). (**c**) The end of the experiment (March). AC, adipocyte; DC, digestive cell; LU, lumen of the midgut diverticulum; SC, secretory cell.

During the overwintering experiment, in both sexes, the midgut was composed of a branched system of diverticula, with the epithelium consisting of digestive cells, secretory cells and adipocytes (Fig. 1). Comparable changes in the structure of the midgut diverticula epithelial cells appeared during the experiment, among which the most prominent feature was the augmented ratio of vacuolized digestive cells, secretory cells and adipocytes (Fig. 1b,c).

### Beginning of the overwintering experiment

**Figure 2:**
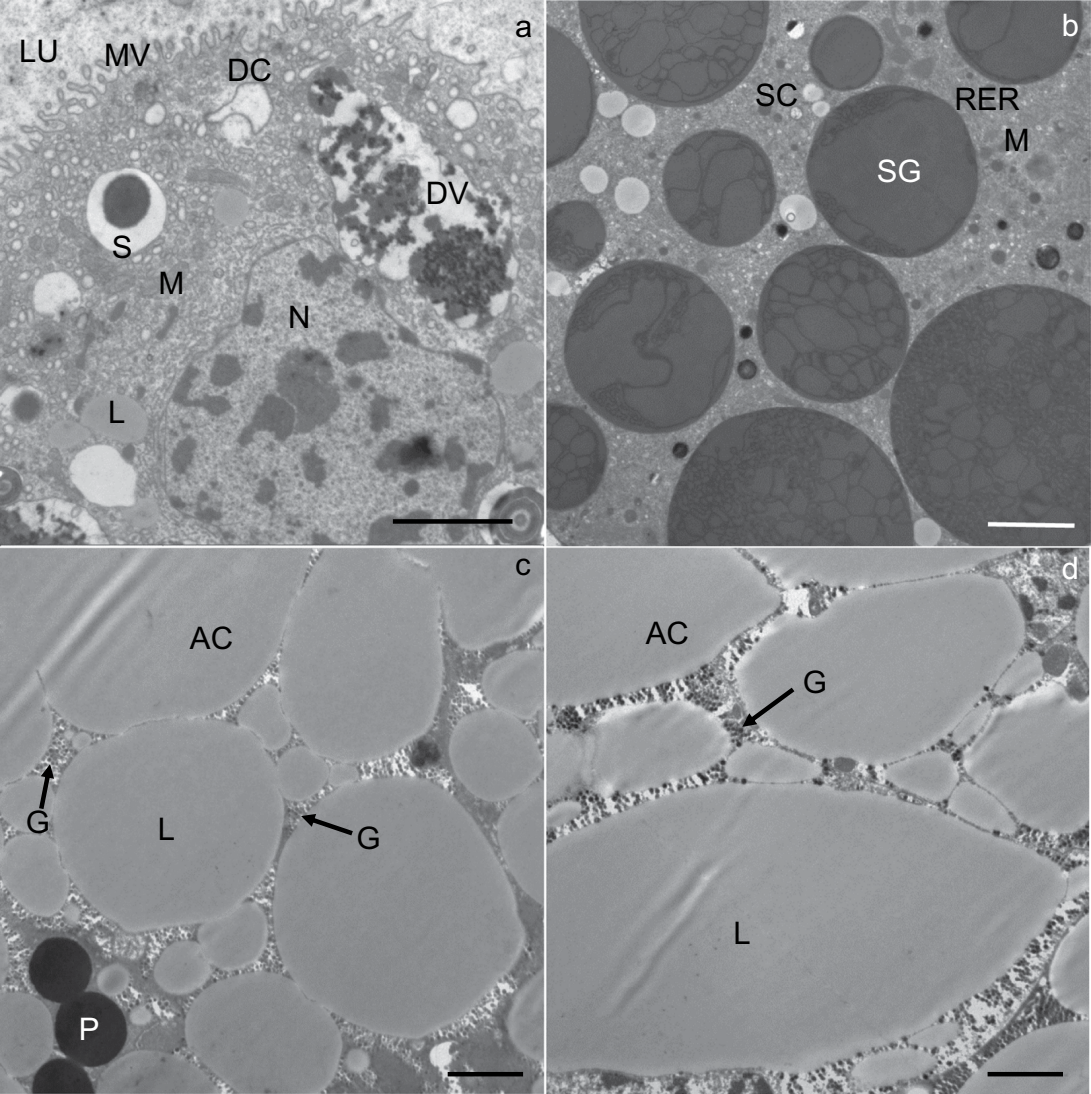
Ultrathin section of the midgut diverticula of *M. menardi* at the beginning of the experiment in November. (**a**) Digestive cell (DC); male. (**b**) Secretory cell (SC); male. (**c**) Adipocyte (AC); male (**d**) Adipocyte (AC); female. DV, digestive vacuole; G, glycogen granules; L, lipid droplet; LU, lumen of the midgut diverticulum; M, mitochondrium; MV, microvilli; N, nucleus; P, protein granulum; RER, rough endoplasmic reticulum; S, spherite; SG, secretory granulum. Scale bars: (**a**,**b**) 2 µm; (**c**,**d**) 1 µm.

The apical plasma membrane of the digestive and secretory cells was differentiated into numerous microvilli projecting into the lumen of the midgut diverticulum (Fig. 2a). The digestive cells were characterized by digestive vacuoles containing material of different electron density (Fig. 2a). Beside digestive vacuoles, located predominantly in the apical part of the cell, the cytoplasm contained mitochondria and spherites. The spherites were round, composed mostly of concentric layers of electron-lucent and electron-dense material, and a membrane (Fig. 6a,b). In some spherites, the material was homogenous and electron-dense, and did not show a laminated structure (Fig. 2a). A round to oval nucleus was located centrally in the cell (Fig. 2a).

The secretory cells were characterized by an abundant rough endoplasmic reticulum (RER) and many electron-dense secretory granules (Fig. 2b). Besides RER and secretory granules, Golgi complexes, mitochondria and spherites were present. In some secretory cells, individual lipid droplets were seen. A round to oval nucleus was located centrally in the cell. In the adipocytes, there were many lipid droplets (Fig. 2c,d), many of them with the longest diameter over 5 µm (Fig. 2d, Table 1).

**Table 1:** Descriptive statistics for lipid droplet diameter and protein granule diameter and glycogen granule abundance in the midgut epithelial cells of *Meta menardi* during winter starvation under control.

	Time frames of starvation experiment	Lipid droplet diameter (μm) (N per sample = 100)	Glycogen abundance (N/μm^2^) (N per sample = 30)	Protein granule diameter (μm) (N per sample = 30)
Sex		Mean ± St.Dev	Mean ± St.Dev	Mean ± St.Dev
		Min–Max	Min–Max	Min–Max
♂	Beginning	3.1 ± 1.7	16.7 ± 2.9	3.6 ± 0.8
		0.7–8.5	10–21	1.9–4.5
	Middle	2.9 ± 1.5	8.5 ± 2.1	3.2 ± 0.7
		0.5–1.8	4–13	1.5–4.5
	End	1.6 ± 0.9	5.2 ± 1.7	2.1 ± 0.7
		0.1–4.3	3–9	0.7–3.2
♀	Beginning	3.5 ± 1.9	19.0 ± 2.6	3.7 ± 0.5
		0.7–9.0	14–24	2.5–4.7
	Middle	2.1 ± 1.0	9.0 ± 2.7	3.1 ± 0.4
		0.3–4.3	5–15	2.3–3.8
	End	1.1 ± 0.4	5.7 ± 1.8	1.8 ± 0.6
		0.1–1.9	2–10	0.6–2.5

Middle - after 58 days, and end - after 118 days of starvation, sample: sex in each time frame.

In numerous adipocytes, the cytoplasm was filled with lipid droplets so that these were in close contact and, consequently, many neighboring lipid droplets were fused (Fig. 2c). Between the lipid droplets, the cytoplasm was locally electron-dense because of numerous glycogen granules (Fig. 2c,d). Besides lipid droplets and glycogen granules, there were mitochondria and some spherites in the adipocytes. Most spherites were composed of concentric layers of electron-lucent and electron-dense material, while some were round or oval and composed of homogenous electron-dense material. In the cytoplasm, protein granules were also seen (Fig. 2c). The nucleus was round to irregular because of the lateral pressure of lipid droplets.

At the beginning of the overwintering experiment, in all cell types, autophagic structures were scant (Table 2).

**Table 2:** Percentage rates of midgut epithelial cells with autophagic structures in *Meta menardi* during overwintering, as observed by TEM. For each sex and time frame, 300 epithelial cells were randomly selected and analysed.

Time frame Sex	Beginning	Middle	End
♂	10	45	65
♀	12	49	71

### Middle of the overwintering experiment

**Figure 3:**
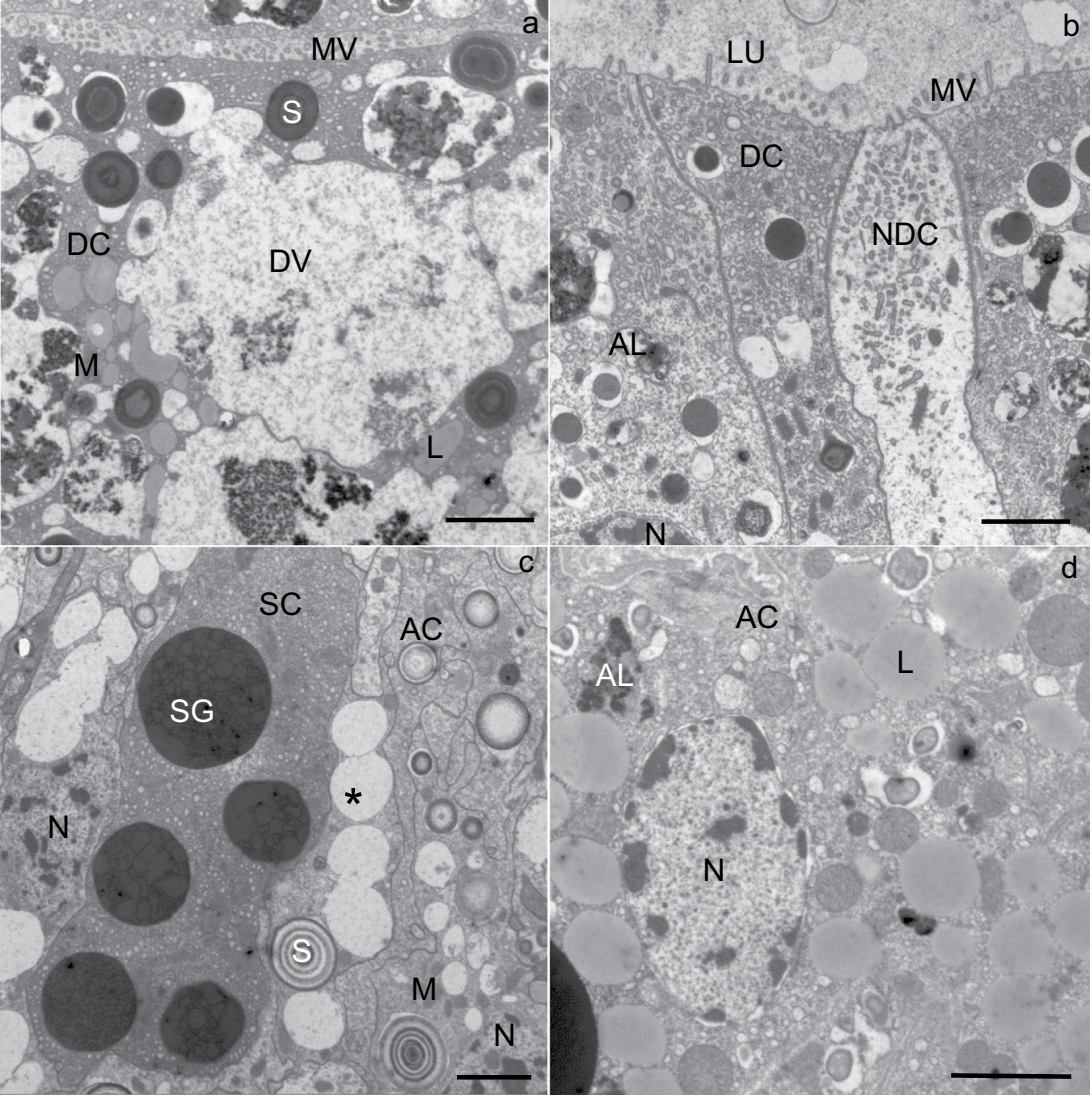
Utrathin section of the midgut diverticula of *M. menardi* in the middle of the experiment in January. (**a**) Digestive cell (DC); female. (**b**) Necrotic digestive cell (NDC); male. (**c**) Secretory cell (SC) and digestive cells (DC); male. (**d**) Adipocyte (AC); female. AL, autolysosome; DV, digestive vacuole; L, lipid droplet; LU, lumen of the midgut diverticulum; M, mitochondrium; MV, microvilli; N, nucleus; S, spherite; SG, secretory granulum; asterisk, exploited spherite. Scale bars: 2 µm.

In all three cell types, the general appearance was comparable to those at the beginning of the experiment, except for a few necrotic digestive cells (Fig. 3b). The apical plasma membrane of the necrotic digestive cells was still differentiated into microvilli (Fig. 3b), the nucleus was oval, but the cytoplasm was electron lucent, containing only a few mitochondria and many small vacuoles (Fig. 3b).

In the cytoplasm of all cell types, autophagic structures were seen (Fig. 3b,d); these were relatively abundant in the digestive cells and adipocytes (Fig. 3b), but rare in the secretory cells (Fig. 3d). Autolysosomes (Fig. 6c,d) and residual bodies were the predominant autophagic structures. Most residual bodies were composed of a material of different electron density, and some additionally contained lipid droplets.

In all the cell types, some spherites showed a change in structure in comparison to those at the beginning of the overwintering experiment; they comprised only one or two concentric layers of electron-dense material and a spherital membrane. In some digestive cells (Fig. 3a), adipocytes (Fig. 3b) and secretory cells (Fig. 3c), the material of the spherites was completely exploited and only the spherital membrane was preserved.

In the adipocytes, the reserve compounds were reduced as compared with those for individuals at the beginning of the overwintering experiment. The longest diameter of lipid droplets measured less than 2 µm (Table 1), and the cytoplasm contained less glycogen than at the beginning of the experiment (Fig. 3d).

### End of the overwintering experiment

**Figure 4:**
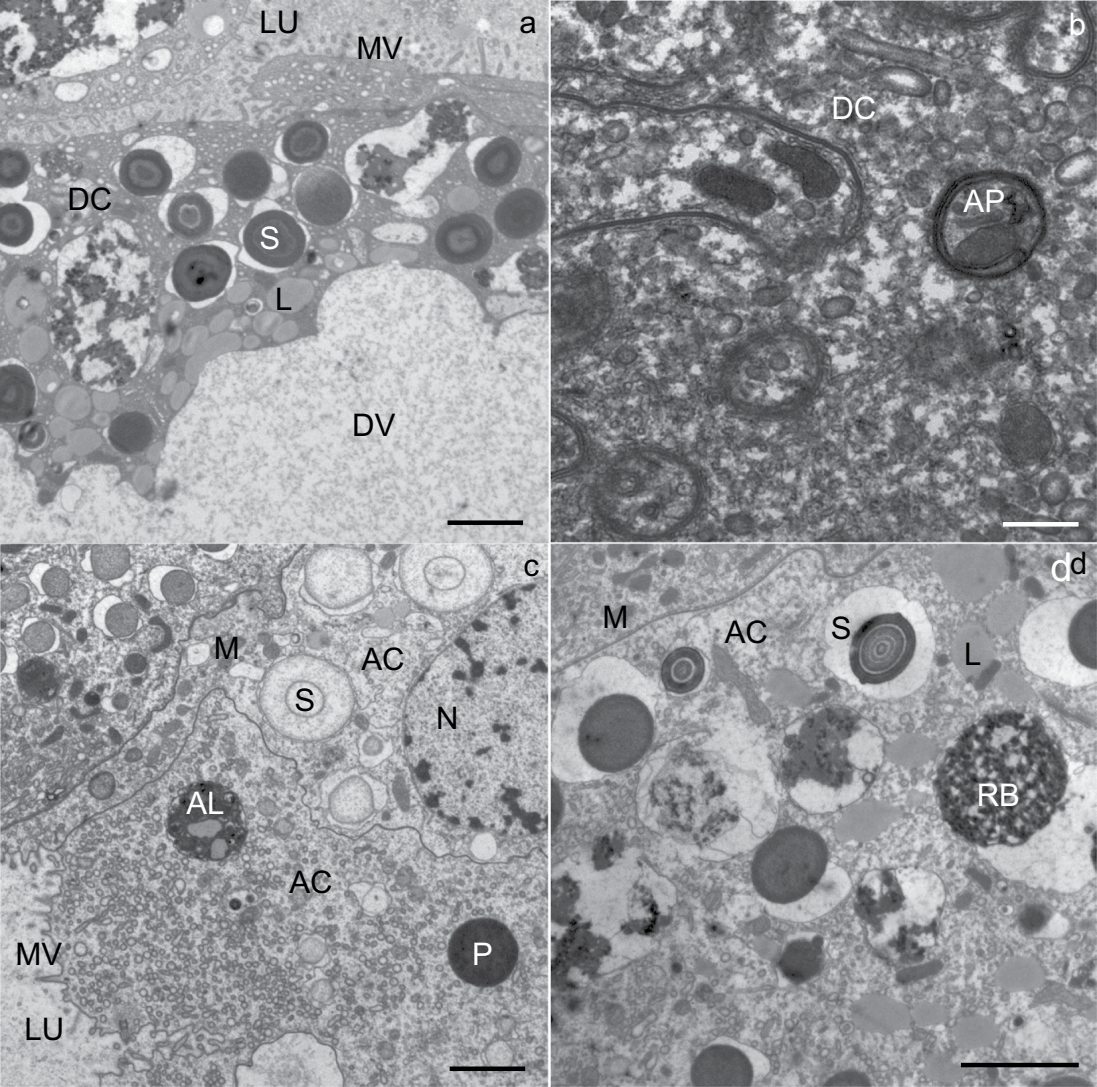
Utrathin section of the midgut diverticula of *Meta menardi* at the end of the experiment in March (male). (**a**,**b**) Digestive cell (DC). (**c**,**d**) Adipocyte (AC). AL, autolysosome; AP, autophagosome; DV, digestive vacuole; L, lipid droplet; LU, lumen of the midgut diverticulum; M, mitochondrium; MV, microvilli; N, nucleus; P, protein granulum; RB, residual body; S, spherite. Scale bars: (**a**,**c**,**d**) 2 µm; (**b**) 500 nm.

**Figure 5:**
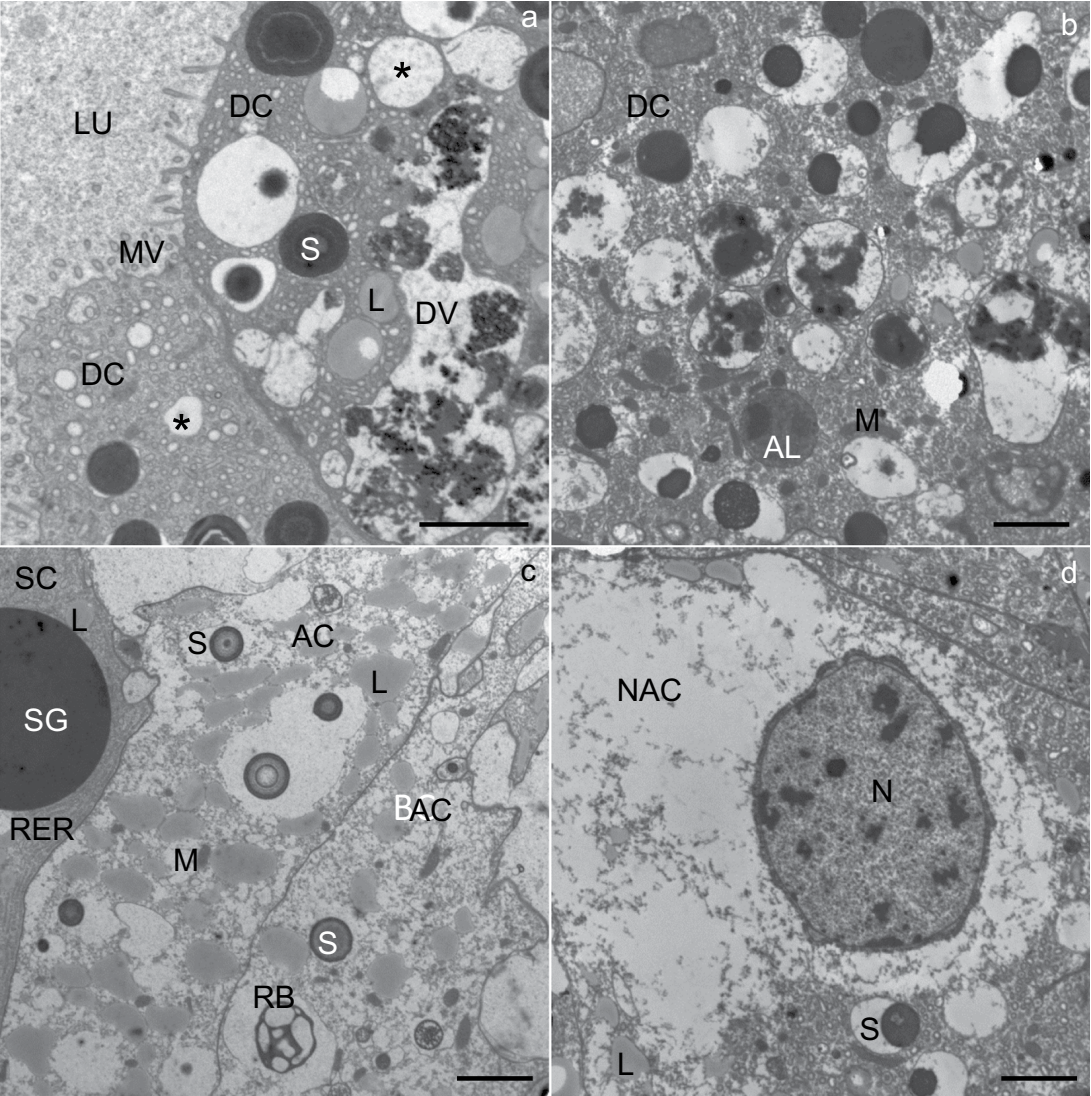
Utrathin section of the midgut diverticula of *Meta menardi* at the end of the experiment in March (female). (**a**,**b**) Digestive cell (DC). (**c**) Secretory cell (SC) and adipocyte (AC). (**d**) Adipocyte (AC). AL, autolysosome; AP, autophagosome; DV, digestive vacuole; L, lipid droplet; LU, lumen of the midgut diverticulum; M, mitochondrium; MV, microvilli; N, nucleus; NAC, necrotic adipocyte; RB, residual body; RER, rough endoplasmic reticulum; S, spherite; SG, secretory granulum; asterisk, exploited spherite. Scale bars: 2 µm.

The structures of the secretory cells, digestive cells and adipocytes were comparable to those in individuals at the beginning of the experiment (Figs 4 and 5). Some digestive cells contained a huge, electron-lucent vacuole and a few smaller vacuoles containing electron-dense material (Fig. 4a). A few digestive cells were necrotic. In some digestive cells, vacuolized cytoplasm was also present (Fig. 5a). A few necrotic digestive cells were characterized by electron-lucent cytoplasm and damaged compartments.

**Figure 6:**
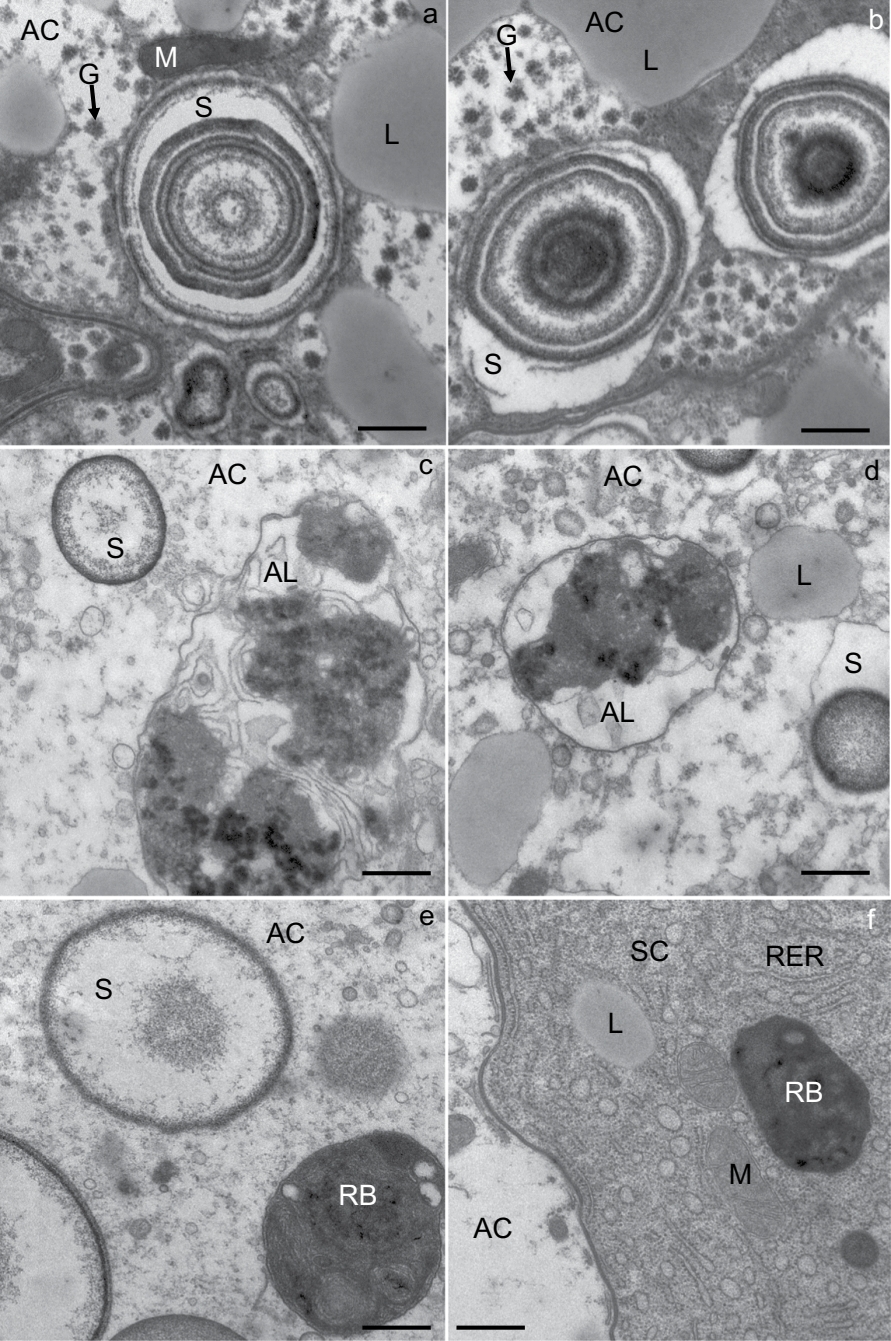
Ultrathin section of the midgut diverticula of *M. menardi* at the beginning of the overwintering experiment in November (**a**: male; **b**: female), in the middle of experiment in January (**c**: male; **d**: female) and at the end of experiment in March (**e**: male; **f**: female). AC, adipocyte; AL, autolysosome; G, glycogen; L, lipid droplet; M, mitochondrium; RB, residual body; RER, rough endoplasmic reticulum; S, spherite; SC, secretory cell. Scale bars: 500 nm.

Autophagic structures were found in all the cell types, but they were most abundant in the digestive cells (Figs 4b and 5b) and adipocytes (Figs 4c,d and 5c). In the digestive cells, there were many autophagosomes (Fig. 4b), autolysosomes (Fig. 5b) and residual bodies. In the adipocytes, as well, various autophagic structures were present; however, residual bodies predominated (Figs 4d and 6e). In a few secretory cells individual autophagosomes, autolysosomes and residual bodies (Fig. 6f) were found.

Many spherites consisted only of one or two concentric layers of electron-dense material and the membrane (Figs 4c and 6e). Some digestive cells and adipocytes contained spherites composed of the membrane only (Figs 4c and 5e).

In the adipocytes, the reserve compounds were reduced in comparison to individuals at the beginning of the experiment; the diameter of lipid droplets diminished (Table 1) and the cytoplasm contained less glycogen than at the beginning of the experiment. Some adipocytes were necrotic (Fig. 5d), with electron-lucent cytoplasm (Fig. 5d), scarce spherites and remnants of the cell compartments.

Figure 6 shows representative micrographs of the main changes in spherite morphology, autophagosomes, residual bodies and glycogen granules between the beginning (Fig. 6a,b), the middle (Fig. 6c,d) and the end (Fig. 6e,f) of the experiment.

### Quantification of autophagic structures

In all three cell types, autophagosomes, autolysosomes and residual bodies were present throughout the experiment. The percentage rates of autophagic cells increased from the beginning until the end of the experiment (Table 2).

### Quantification of reserve lipids, glycogen and proteins

Table 1 shows descriptive values for the lipid droplet diameters, protein granule diameters and glycogen granule abundance in the midgut epithelial cells of *M. menardi* during the winter starvation under control. The GLM factorial ANOVA (with time frames and sex as factors) retrieved significant differences in lipid droplet diameters between males and females and between time frames (Table 3). In males, the use of lipids, according to lipid droplet diameters, was negligible in the first half of the experiment and intensified in the second half. In contrast, in females, the exploitation of lipids was steady during the whole experiment. From the beginning until the middle of overwintering, the average lipid droplet diameter diminished by 0.004 µm/day in males, and by 0.025 µm/day in females, and from the middle until the end of overwintering, by 0.021 µm/day, and by 0.016 µm/day, respectively.

**Table 3:** Two-way ANOVA of lipid droplet diameter, glycogen granule counts and protein granule diameter in the midgut epithelial cells of *Meta menardi* between time frames of the experiment, and sexes. Simple and combined parameters are presented. Significant differences in bold.

	SS	Df	MS	F	p
**Lipid droplet diameter**					
Intercept	333997.2	1	333997.2	1932.64	**<0.0001**
Time frame	38182.5	2	19091.3	110.47	**0.0001**
Sex	1262.2	1	1262.2	7.30	**0.0071**
Time frame *Sex	3910.8	2	1955.4	11.32	**<0.0001**
Error	102308.7	592	172.8		
**Glycogen counts**					
Intercept	20586.8	1	20586.8	3759.16	**<0.0001**
Time frame	4996.2	2	2498.1	456.16	**<0.0001**
Sex	54.5	1	54.5	9.94	**0.0019**
Time frame *Sex	30.6	2	15.3	2.80	0.0638
Error	952.9	174	5.5		
**Protein granule diameter**					
Intercept	1518.4	1	1518.4	3748.46	**<0.0001**
Time frame	87.0	2	43.5	107.40	**<0.0001**
Sex	0.7	1	0.7	1.60	0.2076
Time frame *Sex	1.3	2	0.6	1.54	0.2166
Error	70.5	174	0.4		

During the experiment, differences in glycogen granule abundance were significant between sexes and between time frames (Table 3). In both sexes, the exploitation of glycogen was more intensive in the first half of the experiment (Table 1). Counts of glycogen granules diminished during the experimental starvation; from the beginning until the middle of overwintering, the average counts diminished by 0.14 granule/day in males, and by 0.17 granule/day in females, and from the middle until the end of overwintering, by 0.05 granule/day in both sexes.

During the experiment, there were no significant differences in the diameter of protein granules between sexes, but significant differences did appear between time frames (Table 3). In both sexes, the exploitation of proteins was more intensive in the second half of the experiment (Table 1). From the beginning until the middle of overwintering, the average protein granule diameter diminished by 0.007 µm/day in males, and by 0.010 µm/day in females, and from the middle until the end of overwintering, by 0.018 µm/day, and by 0.022 µm/day, respectively.

## Discussion

In this study, we focused on the ultrastructural changes in the midgut diverticle epithelial cells of *M. menardi* during a four-month winter starvation experiment. These findings enable the completion of the comparative study of responses to starvation in *M. menardi* in critical phases of the life cycle: the beginning and the end of the growth period^35^ with abundant food and winter with scarce food (this study). Our experimental conditions mimic the natural starvation period of *M. menardi* which occurs if they fail to catch prey during wintertime.

The midgut of *M. menardi* consists of a branched system of diverticula, as in other spiders^24,36^ and harvestmen^37^. The epithelium of the midgut diverticula was composed of digestive cells, secretory cells and adipocytes. The ultrastructure of digestive cells and secretory cells was comparable to the ultrastructure of these cells in the spider *Coelotes terrestris*^37,38^, and in the harvestmen *Phalangium opilio*^39^, *Gyas annulatus* and *G. titanus*^33^. The ultrastructure of adipocytes in *M. menardi* was comparable to that in the harvestman *Amilenus aurantiacus*^34^.

At the beginning of the experiment, all the epithelial cells were of normal appearance revealing that all individuals were well fed. Ultrastructural changes gradually appeared during the starvation experiment. As with *M. menardi* starved in spring and autumn^35^, in the middle and at the end of the winter experiment, vacuolized cytoplasm was present in numerous digestive cells. The vacuolization probably originated from degenerated RER, as in columnar cells in the midgut epithelium in starved cockroaches *Periplaneta americana*^40^. In *M. menardi*, a few necrotic digestive cells, all in the initiative phase, were found in the middle and at the end of starvation. The cytoplasm of the necrotic cells was electron-lucent and contained remnant materials of degenerated organelles only, while microvilli could still be recognized. This appearance of the epithelial cells was well comparable to those in starved individuals during the growth period experiments^35^.

In the middle and at the end of the overwintering starvation, autophagosomes, autolysosomes and residual bodies were observed in both sexes in all the cell types: digestive cells, secretory cells and the adipocytes. The appearance of the same autophagic structures has been reported in the midgut diverticules of overwintering harvestmen *Gyas annulatus*^41^ and *Amilenus aurantiacus*^34^, in the fat body and Malpighian tubule cells in overwintering cave crickets *Troglophilus cavicola* and *T. neglectus*^31^ and in herald moths *Scoliopteryx libatrix*^32^. Like in the growth period^35^, in overwintering *M. menardi*, the autophagic structures were often seen in digestive cells and adipocytes, but rarely in secretory cells. Monomeric compounds released from autolysosomes into the cytoplasm serve as building units for various macromolecules, e.g., proteins and carbohydrates, and support the survival of cells during starvation^42,43^. This is what probably happened in the starved *M. menardi*.

In contrast to spring individuals with most spherites exploited^35^, in winter, before the starvation experiment, spherites were numerous, mostly round and composed of tightly packed concentric layers of electron-lucent and electron-dense material, as well as a membrane. In the middle and at the end of the starvation experiment, the material of some spherites was partly to completely exploited. Similar changes in spherites were found in individuals starved in autumn^35^, like in the midgut epithelial cells in overwintering harvestmen *Gyas annulatus*^41^ and *Amilenus aurantiacus*^34^, and in the fat body and Malpighian tubule cells of overwintering cave crickets *Troglophilus cavicola* and *T. neglectus*^31^ and herald moths *Scoliopteryx libatrix*^32^. At the end, in winter-starved *M. menardi*, only the spherite membrane remained of some spherites, while membranes of some others were fused and had formed vacuoles. It seems that the cell vacuolization originated in a large part in this way. The exploited spherite material was−like the material released through autophagy−presumably used to maintain the vital cell processes during the starvation period.

### Quantification of reserve lipids, glycogen and proteins

Like in the growth period^35^, in accordance with our expectations, lipid, glycogen and protein reserves were depleted between the beginning and the end of the winter starvation experiment. In winter, in females, until the middle of the experiment, the diminution of lipid droplet diameter was more intensive than in males. We speculate that females have a more lipid-dependent metabolism as a consequence of oogenesis. In the first half of the experiment, males used glycogen more intensively, probably because, in that time, they were still seeking a mate (cf. ref.^9^) and intensified the use of lipids only after the middle of the experiment. Females can be assumed to use lipids equally over the starvation period because of oogenetic processes. Reserve proteins were probably used to compensate for damage to and loss of constitutive and functional cell proteins.

### Comparison of starvation during growth- and non-growth periods

**Figure 7:**
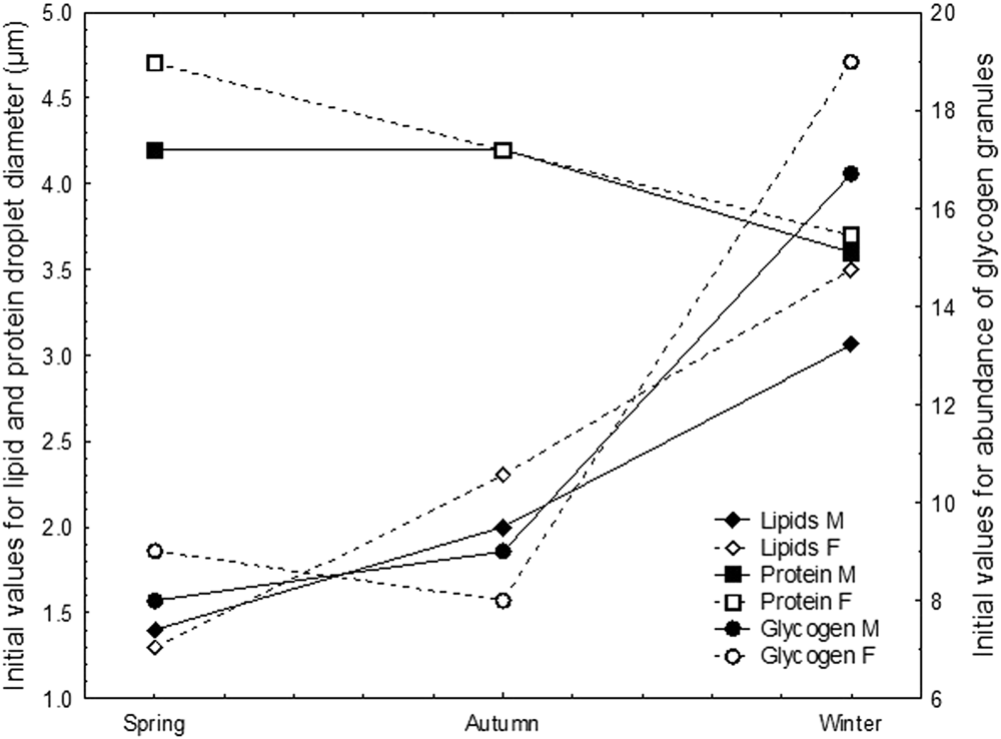
Initial seasonal values for lipid droplet diameter and protein granule diameter and glycogen granule abundance in the midgut epithelial cells of *Meta menardi* during starvation under control over three seasons.

In the present study, we kept *M. menardi* in controlled starvation under the same conditions as in spring and autumn for a previous study^35^. This enables a comparison between the growth- (spring and autumn^35^) and non-growth (winter, this study) periods. The comparison reveals differences in the use of reserve lipids, glycogen and protein stocks. In spring (the post-overwintering phase), adult *M. menardi* are poorly fed because of intensive exploitation of the lipid and glycogen stocks in winter (Fig. 7). In contrast, reserve protein granule diameters are at their largest, possibly because they augment after feeding in the first weeks of spring. Since at the end of overwintering RER becomes more abundant, we speculate that this is the consequence of cells preparing for intensive synthesis of vital proteins at the beginning of the growth period. From autumn (the pre-overwintering phase) until winter (the overwintering phase), reserve lipid and glycogen stocks gradually augment, while reserve protein stocks gradually diminish, probably because proteins are intensively used in growth and developmental processes.

**Figure 8:**
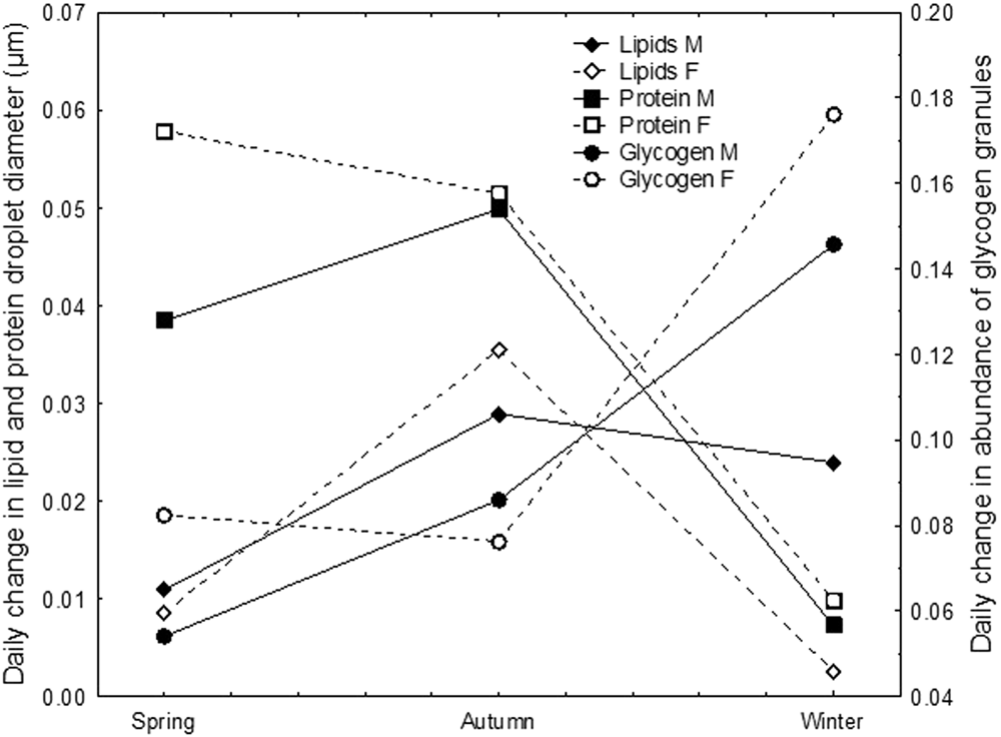
Daily changes of lipid droplet diameter and protein granule diameter and glycogen granule abundance in the midgut epithelial cells of *Meta menardi* during starvation under control over three seasons.

In spring, the average daily use of lipids and glycogen is the lowest and the use of reserve proteins is comparable to the autumn values (Fig. 8). The daily lipid use is highest in autumn. In winter, the use diminishes moderately in males, and conspicuously in females. We assume that this could be because females spend most lipids to form eggs in the period of the autumn-winter shift. We speculate that in autumn, reserve protein use is compensated via protein neogenesis, resulting in a negligible change in protein granule diameters. In winter, in both sexes, glycogen use conspicuously increases, but more in females than in males, which probably balances the deficit in the use of reserve lipids in females. In both sexes, the daily use of reserve proteins is scant, because these are presumably used only in repair processes. During overwintering, *M. menardi* switch from a balanced lipid-glycogen use of reserve stocks during the growing period, to an explicitly glycogen metabolism. We speculate that prior to winter starvation, lipids are used in glycogen production. The glycogen metabolism was reported for the harvestmen *Gyas annulatus*, and enables prompt reaction to changes in the overwintering microhabitat^33,44^. In winter, the glycogen metabolism allows *M. menardi* to have the stand-by readiness to grasp occasional prey, along with other advantages.

## Conclusions

In *M. menardi* starved under control in winter, changes appear in the midgut diverticula epithelial cells, that are characteristic of overwintering processes in many other arthropods. These changes include intensification of autophagy and spherite exploitation and gradual depletion of reserve lipids, glycogen and proteins. All these demonstrate that *M. menardi* is well adapted to survive winter starvation without food until occasional prey becomes available. Thus, *M. menardi* show a general survival pattern featured in many epigean arthropods that undergo programmed winter starvation. In summary, *M. menardi*, in its life cycle begins to renew energy- and nutrient-supplying stocks at the beginning of the growth period in spring, and transitions from a primarily lipid metabolism in autumn, to an explicit glycogen metabolism with sparse use of reserve proteins in winter.

## Material and Methods

The experimental procedure was designed in the same way as during the spring and autumn experimental starvation^35^. We collected 30 adults; 15 males and 15 females in three caves (locality centroid 46°24′55″N, 15°10′31″E; altitudes 600–740 m) in northern Slovenia in November. We divided the spiders for analysis into three groups, with 5 males and 5 females each. Individuals of the second and third groups were held without food in captivity in glasses with wet paper in a refrigerator at 8 °C. The first group was analyzed immediately (November; beginning of the overwintering experiment), the second group in the middle (January), and the third one at the end (March) of the starvation experiment.

### Light and transmission electron microscopy (TEM)

For structural and ultrastructural studies, the midgut diverticula were cut into small pieces and fixed in 2.45% glutaraldehyde and 2.45% paraformaldehyde in a 0.1 M sodium cacodylate buffer (pH 7.4) at room temperature for 3 h and at 4 °C for 14 h, washed in a 0.1 M sodium cacodylate buffer (pH 7.4) at room temperature for 3 h and postfixed with 2% OsO_4_ at room temperature for 2 h. The samples were dehydrated in a graded series of ethanol (50%, 70%, 90%, 96%, 100%, each for 30 minutes at room temperature) and embedded in TAAB epoxy resin (Agar Scientific Ltd., Essex, England). For light microscopy, semi-thin sections (5 μm) were stained with 0.5% toluidine blue in aqueous solution and analysed by a light microscope Nikon Eclipse E800. We used a Nikon DN100 camera. For TEM, ultra-thin sections (75 nm) of the midgut diverticula were transferred to copper grids, stained with uranyl acetate and lead citrate and analyzed by a Zeiss EM 902 transmission electron microscope. In each sex and time frame, the percentage of epithelial cells with autophagic structures was calculated by random counting in 300 midgut epithelium cells. Cells observed at a magnification of 3000x, containing autophagic structures were considered autophagic cells.

### Quantification of reserve lipids and glycogen by TEM

To estimate conditions with respect to these reserve compounds in the midgut epithelial cells during starvation, for each time frame and sex, we measured the diameters of 125 lipid droplets per sample, and counted the glycogen rosettes in 30 1-μm^2^ squares on micrographs.

### Statistical analysis

The data distribution of lipid droplet diameters and protein granule diameters, and the glycogen granule counts were tested for normality using the Kolmogorov-Smirnov test. Since the test did not differ from the normal distribution, two-way ANOVA was used in testing differences between means for sex and time frame. Pattern variabilities of lipid and protein granule diameters, and glycogen granules counts are presented with means plots.

### Ethical Approval and Informed Consent

All the experiments were carried out in accordance with the relevant guidelines.

## Data Availability

The datasets generated during and/or analysed during the current study are available from the corresponding author on reasonable request.
